# War Department: Surgeons General’s Office Circular, No. 3 Report of Surgical Cases in the Army, from 1865 to 1871

**Published:** 1872-03

**Authors:** 


*War Department: Surgeon-General's Office Circular No. 3. Report of Surgical Cases in the Army from* 1865 *to* 1871. By Geo. A. Otis, M.D., Assistant Surgeon U. S. Army.

Surgeon Otis in this Circular has collected, and arranged in order, the Surgical cases occurring in the U. S. army in the years mentioned. He has given an extended account of the more interesting cases, and has illustrated many of them with cuts. Did space allow, we should be very happy to give a more extended notice of these cases, some of which we find very interesting. As Dr. Otis says in his report to the Surgeon-General, "The cases in each category are not sufficiently numerous to warrant any very important generalizations; yet every such contribution must be of value in adding to the mass of facts from which important inferences may be hereafter deduced."

